# A Mid-Infrared Quantum Cascade Laser Ultra-Sensitive Trace Formaldehyde Detection System Based on Improved Dual-Incidence Multipass Gas Cell

**DOI:** 10.3390/s23125643

**Published:** 2023-06-16

**Authors:** Tao Wu, Renzhi Hu, Pinhua Xie, Lijie Zhang, Changjin Hu, Xiaoyan Liu, Jiawei Wang, Liujun Zhong, Jinzhao Tong, Wenqing Liu

**Affiliations:** 1 Key Laboratory of Environment Optics and Technology, Anhui Institute of Optics and Fine Mechanics, Hefei Institutes of Physical Science, Chinese Academy of Sciences, Hefei 230031, China; 2 Graduate School of Science Island Branch, University of Science and Technology of China, Hefei 230026, China; 3 University of Chinese Academy of Sciences, Beijing 100049, China; 4 School of Pharmacy, Anhui Medical University, Hefei 230032, China

**Keywords:** gas sensor, detection of trace HCHO, mid-infrared, dual-incidence multi-pass cell, field campaign, unattended continuous operation

## Abstract

Formaldehyde (HCHO) is a tracer of volatile organic compounds (VOCs), and its concentration has gradually decreased with the reduction in VOC emissions in recent years, which puts forward higher requirements for the detection of trace HCHO. Therefore, a quantum cascade laser (QCL) with a central excitation wavelength of 5.68 μm was applied to detect the trace HCHO under an effective absorption optical pathlength of 67 m. An improved, dual-incidence multi-pass cell, with a simple structure and easy adjustment, was designed to further improve the absorption optical pathlength of the gas. The instrument detection sensitivity of 28 pptv (1σ) was achieved within a 40 s response time. The experimental results show that the developed HCHO detection system is almost unaffected by the cross interference of common atmospheric gases and the change of ambient humidity. Additionally, the instrument was successfully deployed in a field campaign, and it delivered results that correlated well with those of a commercial instrument based on continuous wave cavity ring-down spectroscopy (R^2^ = 0.967), which indicates that the instrument has a good ability to monitor ambient trace HCHO in unattended continuous operation for long periods of time.

## 1. Introduction

HCHO is the most abundant carbonyl compound in the troposphere, which has high activity. It can generate HO_2_ free radicals through photolysis, and then, HO_2_ and NO will react quickly to generate OH free radicals [1]. The photolysis process of HCHO does not consume free radicals, so HCHO is considered an important and unique source of atmospheric free radicals. HCHO is also involved in the generation and removal of RCOOH, peroxyacetyl nitrate (PAN), O_3_, and many other compounds in the atmosphere. Therefore, HCHO has become an important indicator of atmospheric reactivity and a precursor of urban atmospheric aerosol, affecting the oxidation capacity in the troposphere [2,3]. Owing to HCHO’s importance in several key atmospheric processes, investigations on HCHO can help to understand atmospheric chemical mechanisms and validate chemical models [4,5,6]. In addition, HCHO has been identified as a carcinogen by the World Health Organization. Exposure to high levels of HCHO is carcinogenic and genotoxic, which is a considerable concern [7]. Hence, a trace formaldehyde detection with a fast time response is desired for environmental monitoring.

At present, the methods of trace HCHO detection mainly include the chemical method [8], mass spectrometry [9,10], and the spectral detection method. Among them, the spectral detection method has been gradually applied to the trace detection of HCHO because of its high sensitivity, fast response speed, strong selectivity, and non-destructive characteristics [11]. Early trace HCHO spectroscopic measurements focused on the UV–visible band, the technologies commonly used in this band include differential optical absorption spectroscopy (DOAS) [12] and laser-induced fluorescence (LIF) [13], incoherent broadband cavity enhanced absorption spectroscopy (IBBCEAS) [14], etc. Compared with the ultraviolet band, the measurement of the infrared band has a more obvious “fingerprint characteristic”. The technology commonly used in the infrared band mainly includes Fourier transform infrared spectroscopy (FTIR) [15], continuous wave cavity ring-down spectroscopy (CRDS) [16], integrated cavity output spectroscopy (ICOS) [17], tunable diode laser absorption spectroscopy (TDLAS) [18], etc. TDLAS has a compact structure and is easy to integrate. Compared with CRDS, ICOS, and other technologies, TDLAS does not require high-precision mode coupling and has higher stability; hence, TDLAS has attracted extensive attention in the detection of trace HCHO gas.

In the near-infrared band, the measurement of HCHO is mainly focused on the 2*v*_5_ absorption band of 1.7~1.8 μm. However, since the absorption of gas molecules in the near-infrared band corresponds to the transition between atomic energy levels and the vibration spectral band of the pan-frequency laser region of molecular vibration, its absorption spectral line intensity is 2~3 orders of magnitude lower than the fundamental frequency absorption spectral line intensity in the mid-infrared band (*v*_2_ absorption band [19,20] of 5.6~5.85 μm and *v*_4_ absorption band [21,22] of 3.6 μm, corresponding to the transition between molecular rotational and vibrational levels). Accordingly, higher HCHO detection sensitivity can be achieved in the mid-infrared. Early mid-infrared laser sources included the cryogenically cooled lead–salt laser [23,24] and difference-frequency generation (DFG) [25,26]. Working in the mid-infrared in continuous-wave (CW) operation of HCHO detection instruments, based on lead salt, requires refrigeration devices to maintain the light source below 210 K, which greatly reduces the portability of the system [24]. DFG sources have a large footprint and produce mid-IR radiation with low power output [27]. Besides, the common-mode DFG noise structure limits the sensitivity of the HCHO detection device [28]. To suppress the noise structure, the complexity of the system needs to be greatly increased. Therefore, these have been largely superseded by high-performance room-temperature-operation quantum cascade lasers (QCL) and interband cascade lasers (ICL). After that, more and more HCHO detection devices, based on ICL or QCL, were developed. However, there are still some questions about the application of these sensors in high-precision field observation. For example, Li et al. [29] implemented a QCL coupled to an astigmatic Herriott cell [30] with a 36 m effective absorption length. A detection limit of 340 pptv (The unit “pptv” in the paper is equivalent to “pmol/mol”). was attained with an 80 s to 90 s response time. The detection limit indicates a potential application for monitoring in polluted urban environments, but it is poorer than it is required to be for unpolluted tropospheric HCHO measurements. A ppb-level HCHO sensor was developed by Dong et al. [31], with an ICL at 3.6 μm and a novel miniature dense pattern multi-pass cell of 54.6 m, which has a detection limit of 3 ppbv (The unit “ppbv” in the paper is equivalent to “nmol/mol”) for HCHO measurement. With a precision of 1.25 ppbv for direct absorption spectroscopy (DAS) and 0.58 ppbv for wavelength modulation spectroscopy (WMS), the detection limit is limited by the interference fringes due to unwanted etalons. Under the premise of selecting the appropriate spot density, a larger mirror can be used to improve the gas absorption optical pathlength [32], but this will also increase the volume of the system and the rate of air change. In addition to the detection limit, the HCHO sensor is also susceptible to cross interference from common atmospheric gases, such as methane and water vapor [33]. Specially, considering HCHO as a tracer of volatile organic compounds (VOCs), the concentration of HCHO has gradually decreased with the reduction in VOCs emissions in recent years, which puts forward higher requirements for the detection of HCHO. Therefore, to achieve high-precision real-time monitoring of trace HCHO gas in the atmospheric environment in long-term comprehensive field observation activities, further research is carried out in terms of system detection sensitivity and anti-interference ability.

In this study, we report a QCL trace HCHO detection instrument of mid-infrared absorption spectroscopy (HCHO–TDLAS) for ambient HCHO measurements. An improved dual-incidence multi-pass cell was used to increase the absorption optical pathlength. In the *v*_2_ absorption band, an HCHO absorption spectrum line with high absorption intensity and low interference was selected. The short-term comparative observation experiment of HCHO concentration in the atmospheric environment, between HCHO–TDLAS and a commercial HCHO detection instrument based on CRDS technology, shows a good correlation. Furthermore, the instrument was successfully deployed in a long-term field campaign for more than 1 month, and satisfactory results have been obtained.

## 2. Experimental Setup

**Figure 1:**
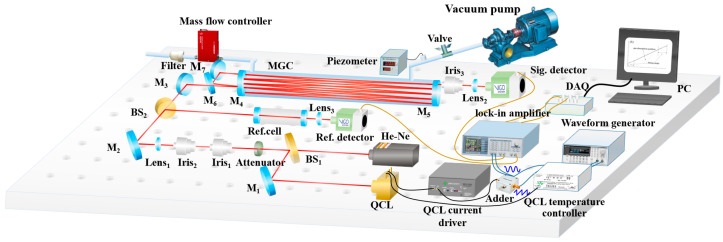
System structure diagram.

The basic principle of TDLAS technology is the Beer–Lambert absorption law. When the laser passes through the absorption cell filled with the gas sample to be measured, the concentration information of the gas can be obtained by measuring the absorption of the gas in a given optical path. Therefore, the concentration of HCHO can be obtained from the attenuation degree of the intensity of the light after the incident light, with an intensity of $I_{0}(v)$, passes through the gas absorption cell with the effective absorption optical path of L. The relationship between them is as follows:

(1) $$I_{t}\left(v\right)=I_{0}\left(v\right)e^{-\alpha (v)L}$$

where v is the frequency of laser and α(v) is the absorption coefficient of the sample gas. When the wavelength modulation scheme was adopted, the inversion of the gas concentration in the actual measurement could be realized by calibrating the harmonic signal and the concentration of HCHO. Based on this principle, we built a TDLAS system for trace HCHO detection. The detection system was mainly divided into the optical path part, circuit part, and signal processing part, as shown in Figure 1.

The light source is a customized QCL (ADTECH, HL-19-80C) with a center wavelength of 5.68 μm. The QCL is fixed on a cooling base (water-cooled) and maintained at about 27 °C by a built-in high stability temperature controller and a thermoelectric cooler (TEC). The wavelength of the QCL was adjusted by changing the output current by superimposing a modulated waveform (generated by a waveform generator) on the current-driven DC bias. The visible red light emitted by the He–Ne laser was used as an indicator light. By adjusting beam splitter BS_1_ and reflector M_1_, the middle infrared light emitted by QCL overlapped with the visible red light. Then, the middle infrared light passes through a mid-infrared attenuator to suppress feedback light caused by the optical components in the optical path. By measuring the beam waist parameters of the middle infrared light source and conducting the Zemax simulation, the parameters of the lens used to match the beam waist of the light source and multi-pass cell were calculated. Based on the calculation results and the fine adjustments made during the experiment, interference noise between multi-pass cell spots was effectively suppressed. Then, the middle infrared light was divided into two beams by a beam splitter (BS_2_), one of which enters the reference cell, and the other enters the multi-pass cell. The reference light (from the reference cell) and the signal light (from the multi-pass cell) passed through the focusing lenses and were, respectively, received by HgCdTe detectors (VIGO, PVI-4TE-6) with thermoelectric cooling (TEC). The data collected by the acquisition card (NI, USB-6361) were processed and analyzed online, by the upper computer, to realize the unattended long-term online monitoring of HCHO concentration. The air inlet of the multi-pass cell was connected to the mass flow controller (SEVENSTOR, CS200) through a PTFE pipe, and the air outlet was connected to the vacuum pump (Edwards, nXDS10i) through a PTFE pipe and an air valve. When measuring the concentration of HCHO, the gas to be measured was pumped into the multi-pass cell by a vacuum pump, the mass flow controller was used to adjust the inlet flow of the multi-pass cell, and the air valve was used to control the pressure of the multi-pass cell. A three-way solenoid valve was used to switch between zero air and the gas to be measured to realize the deduction of the background signal. When the wavelength modulation scheme was adopted, the low-frequency sawtooth wave and high-frequency sine wave, generated by the waveform generator, were superimposed and input to the driving circuit to realize the tuning of the light source. The second harmonic demodulation of the measured signal and reference signal was carried out by the double-lock-in amplifier (SINE SCIENTIFIC INSTRUMENTS, OE1022D). The reference signal was used to lock the position of the absorption peak and correct the measurement result when the wavelength drift occurs.

## 3. Discussion and Results

### 3.1. Spectral Line Selection

It is necessary to select appropriate absorption spectral lines for gas concentration detection using absorption spectrum technology. When selecting the absorption spectral lines of gas, three principles are generally followed: (a) The selected absorption spectral lines should have a sufficient intensity of absorption to ensure that the gas can be measured under the target detection sensitivity; (b) the interference of absorption lines from other molecules—and its own absorption lines at non-target wavelengths—should be avoided, or the complete absorption line shape cannot be obtained and the measurement results will be affected; (c) try to ensure that the edge absorption of water vapor at the selected absorption spectrum line is not too serious to prevent the absorption saturation of water vapor at this wavelength.

The HCHO detection spectral lines in the mid-infrared band are mainly concentrated in the *v*_4_ and *v*_2_ absorption bands. Considering the absorption intensity and gas interference, the *v*_2_ absorption band of 1759.73 cm^−1^ was selected, in this study, after comparing the different HCHO absorption lines of the *v*_2_ and *v*_4_ absorption bands, and the line strength of the absorption line reached 6.02 × 10^−20^ cm/molecule at 25 °C. Moreover, it is not disturbed by common atmospheric gases under specific pressure, and it has higher applicability than other absorption spectra in actual field observation activities. In summary, QCLs with a central wavelength of 5.68 μm were used as the light source of the detection system in this study.

**Figure 2:**
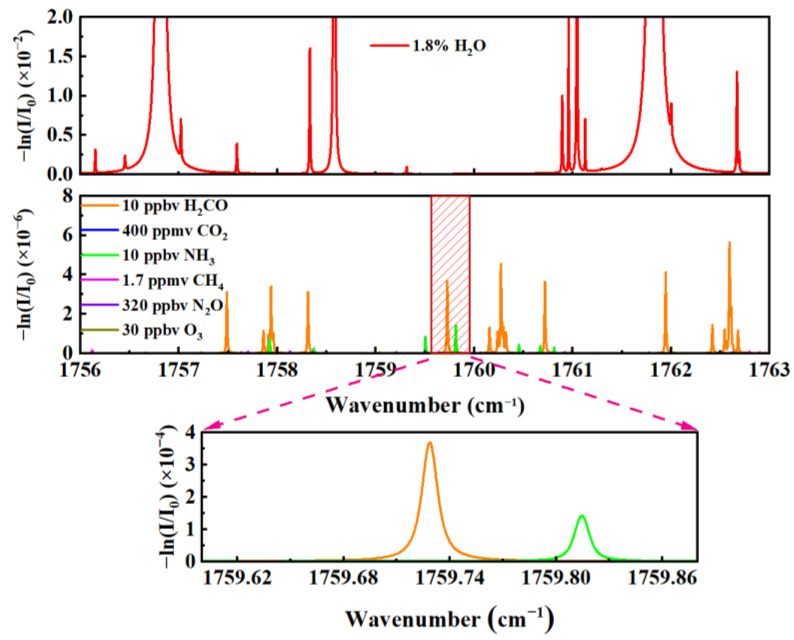
HITRAN database simulation of absorption spectra of HCHO and, potentially, interfering gases in the atmosphere within the tunable range of the QCL.

The HITRAN database was used to analyze HCHO at 10 ppbv and standard air (400 ppmv CO_2_, 10 ppbv NH_3_, 1.7 ppmv CH_4_, 320 ppbv N_2_O, and 30 ppbv O_3_, 1.8% H_2_O) in the spectral range of 1756 cm^−1^–1763 cm^−1^, and the simulation results are shown in Figure 2. The pressure selected for the simulation is 30 Torr and the temperature is 298.15 K. The simulation results show that the absorption lines near the wavelength of 1759.73 cm^−1^ are mainly HCHO and NH_3_, which are hardly affected by other potential interfering gases in the atmosphere. Moreover, under the simulation conditions, the absorption lines of NH_3_ and HCHO can be separated well, and the absorption of HCHO is almost not affected by NH_3_ absorption. Therefore, the gas concentration can be measured more accurately at the target spectral line of 1759.73 cm^−1^.

### 3.2. Multi-Pass Cell Design

**Figure 3:**
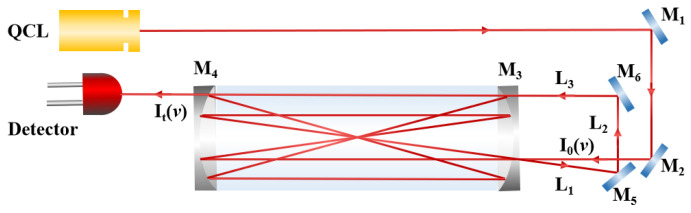
Schematic diagram of the dual-incidence optical path structure.

In the TDLAS gas detection system, the two most important factors that limit its detection sensitivity are the effective absorption optical pathlength of gas and the interference noise between the light spots. In general, it is desirable to achieve a long absorption optical pathlength in a small multi-pass cell. On the premise of making the spots as evenly distributed as possible by beam waist matching, improving the utilization rate of the mirror is a very effective way to ensure low noise and a long absorption optical pathlength. The traditional Herriott multi-pass cell has a stable structure and has been widely used [34], but because their light spots can only be distributed in a single circle on the mirror (taking the distance from the hole to the center of the mirror as the radius), the utilization rate of the mirror is low, which restricts the effective absorption optical pathlength of the multi-pass cell. Therefore, some research groups have proposed new multi-pass cell structures and applied them to the trace detection of HCHO, such as the astigmatic multi-pass cell, the dense spot pattern multi-pass cell, etc. The astigmatic multi-pass cell structure forces the light spot to distribute in a “Lissajous” pattern, due to the non-rotational symmetry of the mirror’s curvature radius, effectively improving mirror utilization. However, the non-uniform distribution of specular spots may adversely impact the detection sensitivity of the system [33,35]. In addition, since the processing of astigmatic mirror is relatively complicated, it is difficult to guarantee the accuracy of the mirror. The dense spot pattern multi-pass cell can effectively enhance mirror utilization, but the off-axis aberration induced by the large angle of light incidence will cause spot deformation [36], so it is necessary to use a larger diameter mirror, which will lead to the increase in the volume of the multi-pass cell [32]. Therefore, we have made some improvements on the basis of the traditional Herriott multi-pass cell [37] and designed a novel multi-pass cell structure. The designed multi-pass cell not only possesses the advantages of a traditional Herriott multi-pass cell, such as excellent stability, absence of spot distortion, and uniform spot distribution, but also enables dual-circle spot distribution and significantly enhances mirror utilization efficiency. Besides, the multi-pass cell has a simple structure and a low cost as well. The dual-incidence is taken as an example here. The schematic diagram of the dual-incidence multi-pass cell, designed in this study, is shown in Figure 3.

**Figure 4:**
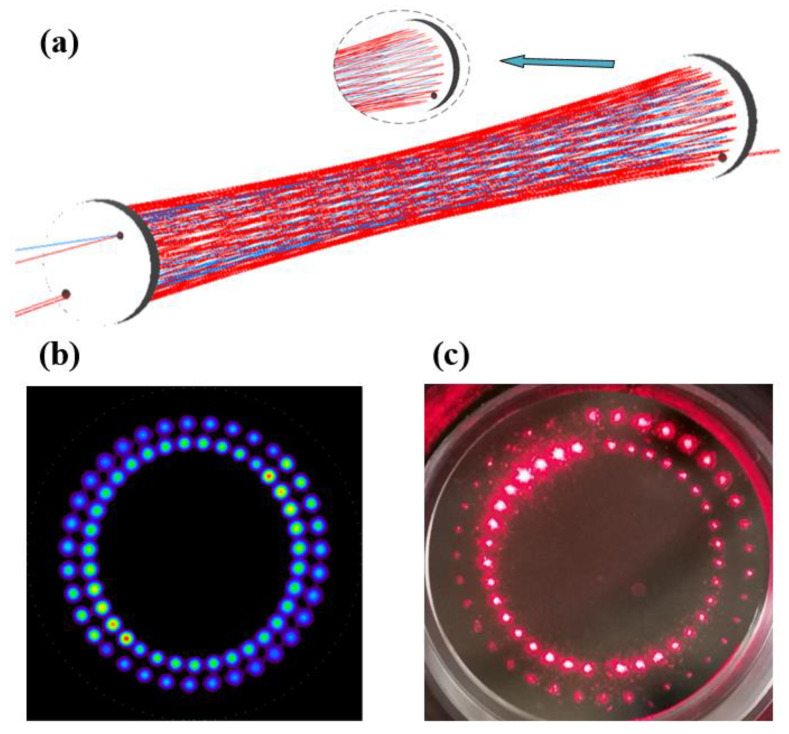
Dual-incidence optical path: (**a**) TracePro simulation optical path; (**b**) TracePro simulation light spot distribution; (**c**) actual light spot distribution.

The dual-incidence multi-pass cell is made up of two spherical mirrors (with a curvature radius of 0.5 m and diameter of 2 inches), and it has a base length of 47.8 cm and a volume of 0.8 L. The spherical mirrors in the multi-pass cell were coated with a silver film to make their reflectivity at 5.68μm reach 0.97. To achieve dual-incidence, the near mirror was perforated with a small hole at the radius of 17 mm and 20 mm, and the diameter of the small hole is 3.2 mm and 4 mm, respectively. The distal mirror is a common curved mirror. TracePro simulation software was used to simulate the dual-incidence optical path, and the results were verified by experiments, as shown in Figure 4.

The effective optical pathlength of the multi-pass cell can be calculated by the actual number of light spots distributed on the mirror’s surface, as demonstrated in Equation (2).

(2) $$L_{\mathrm{eff}}=2\cdot m\cdot L_{\mathrm{base}}$$

$L_{\mathrm{eff}}$ is the effective absorption optical pathlength, $L_{\mathrm{base}}$ is the base length of the multi-pass cell, and m represents the number of spots distributed on a single mirror’s surface. The spot diameter of the mid-infrared beam is about 2.6 mm. As the number of spots distributed on the surface of a single mirror of the proposed dual-incidence multi-pass cell is 70, the effective absorption optical pathlength that can be realized is 67 m. It can be seen that the effective absorption optical pathlength of the dual-incidence multi-pass cell is twice that of the traditional Herriott multi-pass cell at the same volume.

### 3.3. Background Subtraction and Wavelet Denoising

When the 2f-WMS sensor configuration was adopted, a 50 kHz sine wave was added to the 200 Hz sawtooth wave generated from the function generator, so the QCL was tuned around the wavelength of 5.68 μm. The signal was demodulated (second-harmonic, 2f detection) with the lock-in amplifier, with a time constant of 30 μs. The demodulated signal was recorded by a personal computer via a DAQ card. The inlet flow rate of the tank was set to 600 mL/min by a mass flow meter, and the pressure in the multi-pass cell was maintained, at 30 Torr, by a regulating valve in front of the vortex dry pump. High-purity N_2_ and cylinder HCHO gas (Maotoo Inc., Shanghai, China) were mixed and diluted by a multi-component dynamic gas distributor (NIM, MF-5B) to provide a stable HCHO gas mixture for laboratory measurement.

**Figure 5:**
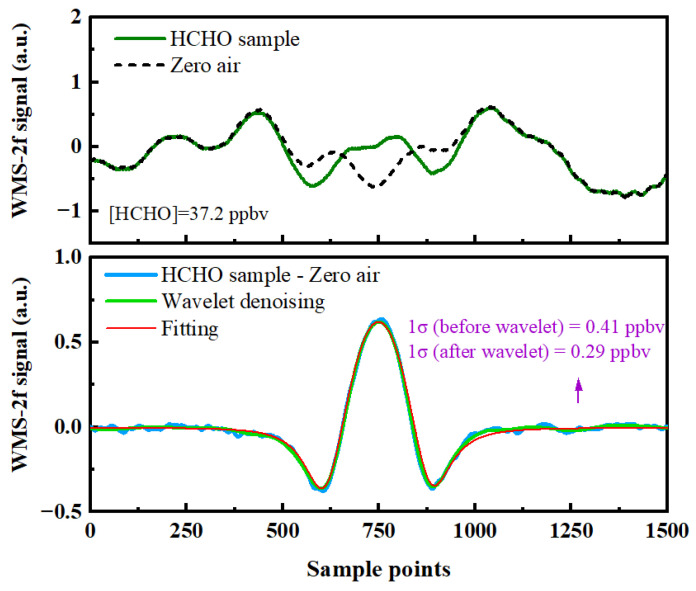
The 2f absorption signal of HCHO with 37.2 ppbv concentration.

In this study, the light source and the beam waist of the multi-pass cell were matched. Although the multi-pass cell mirror interference between the speckle noise could be restrained effectively, the interference fringes may still exist when the laser beam transmits through individual optical elements, such as windows and lenses, or free-space path separated by the surfaces of different optical elements. While fine-tuning the optical element can weaken the interference, it is difficult to remove it from the system effectively and completely. Dual-beam subtraction or zero-nitrogen subtraction methods are normally used to solve the fringe limitation by subtracting a reference spectrum [35]. The zero-air background subtraction method was adopted in this study. In addition, the modulation amplitude and modulation phase were optimized to obtain the optimal second harmonic signal. The acquisition time of each 2f signal was 1 s, as shown in Figure 5.

The upper panel of Figure 5 is the 2f signal demodulated by the lock-in amplifier after gas absorption and 2f of zero air. It can be clearly observed that there is a background signal. To further improve the spectral signal-to-noise ratio, based on the background subtraction method, a digital signal processing algorithm, based on wavelet transform (WT), was used for spectral noise reduction, which operates in both time and frequency domains to obtain a low-frequency profile and high-frequency details of the signal. For the wavelet denoising algorithm, soft thresholding and hard thresholding are commonly employed thresholding methods. The soft thresholding operation consists of setting the wavelet coefficients smaller than the threshold to zero and shrinking the others to zero. The hard thresholding operation keeps the amplitude constant before and after denoising, but it might induce some Gibbs oscillation at the edges due to its discontinuity [38,39]. Compared to the hard thresholding method, the soft thresholding method demonstrates superior smoothing effects. Thus, the soft threshold strategy of a discrete Symlet wavelet basis, with symmetry, was adopted in this study. However, excessive decomposition levels in the soft thresholding method may lead to signal distortion and amplitude reduction. Therefore, the final decomposition level after optimization was set to 7, where the SNR is optimal and the peak value does not decrease significantly. The soft thresholding strategy of a discrete Symlet wavelet basis, with symmetry, was adopted in this study. The lower panel of Figure 5 shows the 2f signal after the subtraction of the background signal, the harmonic signal after wavelet denoising, and the 2f signal obtained by simulation. After background subtraction, an obvious HCHO absorption 2f signal can be obtained. The detection sensitivity is increased from 0.41 ppbv (1σ) to 0.29 ppbv (1σ) by using the wavelet transform digital signal spectral denoising algorithm, and the harmonic signal is in good agreement with the simulation results.

### 3.4. Concentration Calibration

**Figure 6:**
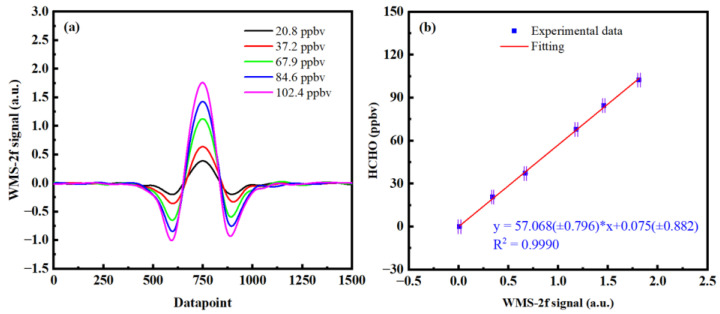
Calibration of the wavelength-modulated 2f signal peak: (**a**) 2f signal at different concentrations; (**b**) calibration fitting of 2f peak to concentrations.

In the actual measurement, the peak intensity of 2f is correlated with the measured HCHO concentration, showing a linear relationship within a certain range. Therefore, before using wavelength modulation technology to detect the HCHO concentration, the diluted stable HCHO mixture with known concentration should be used to calibrate the relationship between the 2f signal peak value and HCHO concentration to obtain the linear relationship between them. When HCHO was measured, the peak voltage of the 2f signal and the fitting formula were used to invert the concentration of HCHO. High-purity N_2_ and cylinder HCHO gas were mixed and diluted evenly, by the gas distributor, to obtain different concentrations of HCHO for calibration, and the calibration results are shown in Figure 6. It can be seen that there is a good linear relationship between the amplitude of WMS–2f and the concentration (correlation coefficient R^2^ is 0.9990), indicating that the developed WMS–HCHO detector meets the requirements. It should be noted that wavelength drift will cause deviation in the measurement of HCHO concentration, so the wavelength drift correction method (described in Section 3.5) was adopted in the calibration of Figure 6.

### 3.5. Wavelength Drift Correction

**Figure 7:**
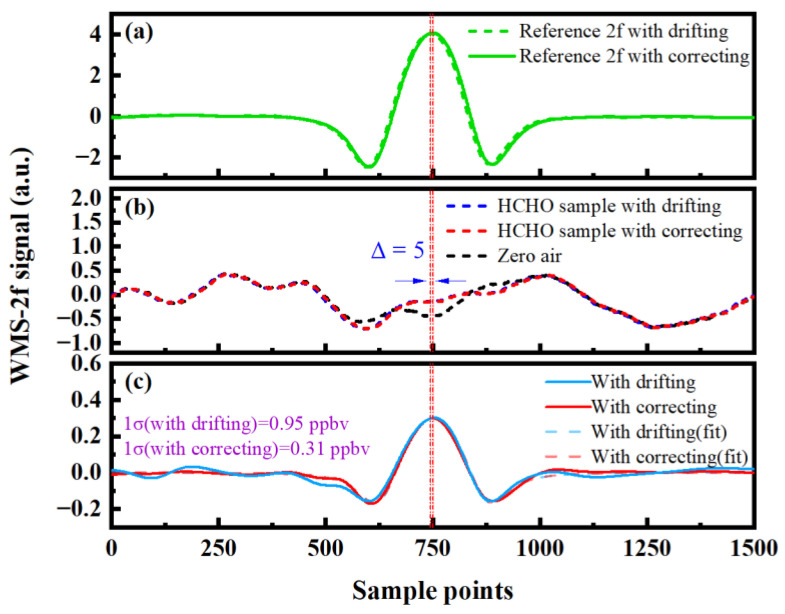
Signal comparison before and after the correction of the wavelength drift position: (**a**) reference 2f signal; (**b**) signal and background; (**c**) 2f signal with background subtraction.

In the actual measurement, the laser wavelength of the light source will drift with the change of time, and the reasons for the drift include the change of the driving current and temperature. The wavelength shift of the laser will lead to a relative offset between the signal and the background. In this case, the shift of peak position between them, during background subtraction, will increase the concentration variability and the measuring error of the measured HCHO concentration. To solve this problem, a wavelength drift correction method was proposed, which is different from the electrical modulation scheme that adopts PID feedback technology to regulate the driving current of the light source, to lock the location of the absorption peak [29]. First, the high concentration of HCHO gas was filled into the reference cell, the transmitted signal light of the reference cell was converted into an electrical signal through the photodetector, and then, it was demodulated by double-channel phase-lock. The parameters of the lock-in amplifiers of the reference path and the measurement path were set to be the same so that the peak positions of the 2f signals of the reference signal and the measurement signal were also consistent. For the 2f signal with a high HCHO concentration in the reference path, it is easy to search for its peak through the software program. A fixed point position was set as the reference point, and the peak position of the measurement signal was corrected, in real-time, according to the deviation of the coordinate points between the reference signal and the reference point, so the errors caused by wavelength drift were suppressed. The 2f absorption signal of HCHO gas, with 17.0 ppbv concentration configured by the dynamic gas distributor, is shown in Figure 7. After a period of measurement, it is evident, from the reference signal in Figure 7a, that the central wavelength of the laser has shifted, indicating that the HCHO absorption signal has also shifted relative to the background signal. Figure 7b shows the HCHO absorbed signal and background signal, and Figure 7c shows the 2f absorbed signal with background subtraction. In the measurement process, the HCHO concentration, corresponding to the fitting standard deviation of the 2f signal, with background subtraction after the wavelength shift, is 0.95 ppbv. After calibrating the position of the 2f absorption signal, the HCHO concentration corresponding to the fitting standard deviation is 0.31 ppbv. It can be seen that the noise of the second harmonic signal, deducted from the background after correction, is smaller. To summarize, the wavelength shift correction method adopted in this study is simpler to implement, and it does not need to add an additional circuit, which can successfully suppress the measurement error caused by wavelength position drift.

### 3.6. Performance Evaluation

**Figure 8:**
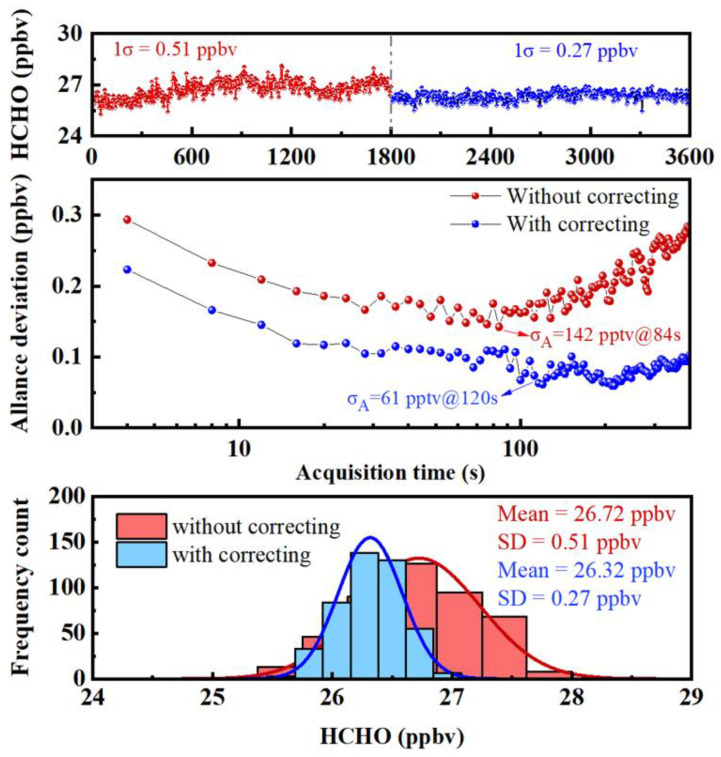
Allan variance of trace HCHO detector.

Allan variance was used to evaluate the detection sensitivity of the trace HCHO sensor before and after the wavelength shift correction method was applied under the WMS scheme, and the results are shown in Figure 8. Under the data acquisition time of 4 s, the detection sensitivity of the system was 142 pptv (1σ) at an average time of 84 s without wavelength shift correction, and the detection sensitivity of 61 pptv (1σ) can be obtained at an average time of 120 s with the wavelength shift correction method. The lower panel of Figure 8 shows the frequency distribution histogram of the HCHO concentration series. The distribution histograms measured before and after using the wavelength shift correction method both show Gaussian distribution, and the standard deviations of the measured HCHO concentration series are 0.51 ppbv and 0.27 ppbv, respectively. For the detection system, uncertainty mainly includes the following three aspects: (1) the uncertainty of the HCHO absorption line strength was ±2.3% (*u*_1_); (2) the uncertainty caused by linear fitting between the second harmonic peak and HCHO concentration is 1.4% (*u*_2_); (3) the uncertainty of the standard gas of HCHO is ±2% (*u*_3_). By using the calculation formula of uncertainty ($\pm \sqrt{u_{1}+u_{2}+u_{3}}$), the uncertainty of the HCHO detection system can be obtained as ±3.4%.

### 3.7. Application of Kalman Adaptive Filtering in Concentration Extraction

According to Allan, variance can assess the detection sensitivity of the system under different average times. The Allan variance defines the optimal averaging time for the gas analyzer, which minimizes the shot-to-shot variability of concentration measurements. Once this averaging time has been chosen, further detection sensitivity improvements can be achieved through the use of the filtering technique. While numerous filtering techniques can be applied to the post-processing of gas concentration measurements, only a few successful techniques have been identified that allow efficient on-line filtering of concentration measurements. A direct technique, which can be applied in real-time, is the simple averaging of previous n measurements. However, the application of the Kalman filter has more obvious advantages [40]. By using a recursive program with the parameter $z_{k}$ (measured value at the time k) and the parameter ${\hat{\delta }}_{k}^{-}$ (predicted value at the time k), the “true rate” ${\hat{\delta }}_{k}$, at the time k, can be predicted. The expression is shown in Equation (3):

(3) $${\hat{\delta }}_{k}={\hat{\delta }}_{k}^{-}+K_{k}{{(z}_{k}-\hat{\delta }}_{k}^{-})$$

**Figure 9:**
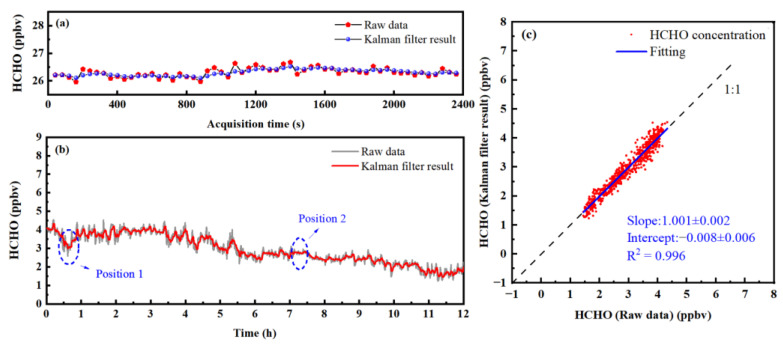
Application of the Kalman filter in HCHO concentration measurement: (**a**) Kalman filtering of standard gases; (**b**) Kalman filtering of concentration series of the outdoor HCHO gas; (**c**) correlation analysis before and after Kalman filtering.

In the equation above, $K_{k}$ represents the Kalman gain, which is associated with both instrument measurement noise ($\sigma_{v}$) and true concentration variability ($\sigma_{w}$). The ratio of $\sigma_{v}^{2}/\sigma_{w}^{2}$, denoted as ρ, is a constant parameter utilized for filter tuning. When applying the Kalman filter for gas concentration extraction, it is customary to estimate the standard deviation of previous sample concentrations as the value of $\sigma_{v}$. For low values of ρ, the filtering efficacy in eliminating shot-to-shot variation is reduced. However, if ρ is too high, the filtered outcome will lag behind actual concentration changes. Therefore, after comprehensive consideration, a value of 20 was set for ρ. For the measured concentration series of the HCHO standard gas with an average time of 40 s, Kalman filtering was performed, and the results are shown in Figure 9a. After Kalman filtering, the values of the concentration variability are reduced from 111 pptv (1σ) to 28 pptv (1σ), and the values are improved by about 4 times. Outdoor HCHO concentration values were measured continuously for 12 h, with an average time of 40 s. The Kalman filtering algorithm was used to process the measured concentration series of the outdoor HCHO gas, and the results are shown in Figure 9b. It can be seen, from position 1 in Figure 9b, that the concentration of HCHO gas extracted by the Kalman filter algorithm effectively follows the change of the true HCHO concentration and can reflect the small change of HCHO gas concentration. In addition, a relatively smooth sequence segment can be obtained when the actual concentration of HCHO gas is stable (refer to position 2 in Figure 9b). The correlation analysis between the original concentration sequence of the HCHO gas and the Kalman filtering results was carried out to further demonstrate the ability to follow true gas concentration, as shown in Figure 9c. The correlation coefficient (R^2^) of the two concentration sequences is 0.996, the slope is 1.001, and the intercept is 0.008. The Kalman filtering results have a high correlation before filtering, indicating that the Kalman filtering method can closely follow the true concentration changes.

### 3.8. Comparison of Detection Sensitivity

The trace HCHO detection system reported in this study adopted an improved dual-incidence multi-pass cell, and the interference phenomenon in the multi-pass pool was suppressed by the beam waist matching. The wavelength drift correction and Kalman adaptive filtering technique were adopted to achieve excellent detection performance. Table 1 shows the comparison of the performance of some TDLAS systems that have been reported.

### 3.9. Laboratory Testing and Analysis

The anti-interference ability of the HCHO detection system and the real-time monitoring ability of atmospheric HCHO were tested and analyzed in the laboratory to ensure that stable and high-precision measurement can be carried out in long-term comprehensive field observation.

#### 3.9.1. Analysis of the Cross Interference of HCHO Adjacent Gas Absorption Lines

**Figure 10:**
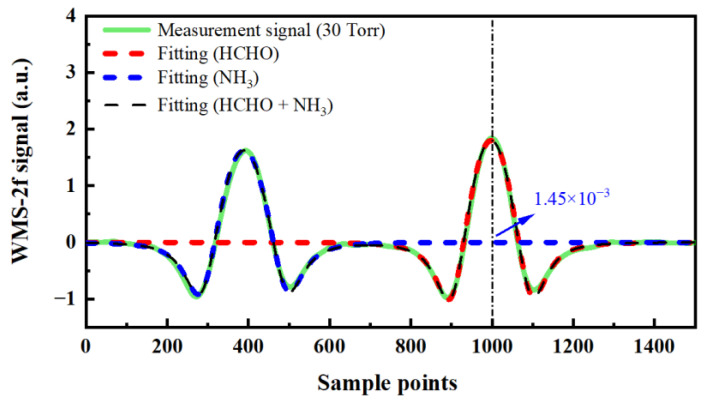
The second harmonic absorption signal of NH_3_ and HCHO at 30 Torr pressure.

In this study, there is an adjacent NH_3_ absorption line near the selected HCHO absorption line. According to the gas absorption simulation results in the HITRAN database mentioned in Section 3.1, the HCHO and NH_3_ absorption lines can be separated under the detection pressure of 30 Torr. To verify the interference of NH_3_, the mixed gas of HCHO (100 ppbv) and NH_3_ (300 ppbv) was measured at 30 Torr detection pressure. The measured 2f signal and fitting results are shown in Figure 10. The concentration of the HCHO, corresponding to the voltage value at the HCHO absorption peak position of the 2f signal of 300 ppbv NH_3_, is 83 pptv. The concentration of NH_3_ in the ambient atmosphere is generally from several ppbv to tens of ppbv. Taking NH_3_ of 30 ppbv as an example, according to the linear relationship between gas concentration and 2f signal amplitude, the HCHO concentration corresponding to the voltage value of the 2f signal of NH_3_ at the HCHO absorption peak position is about 8 pptv, which has little influence on the measurement of atmospheric HCHO in the field campaign.

#### 3.9.2. Analysis of the Influence of Environmental Humidity

**Figure 11:**
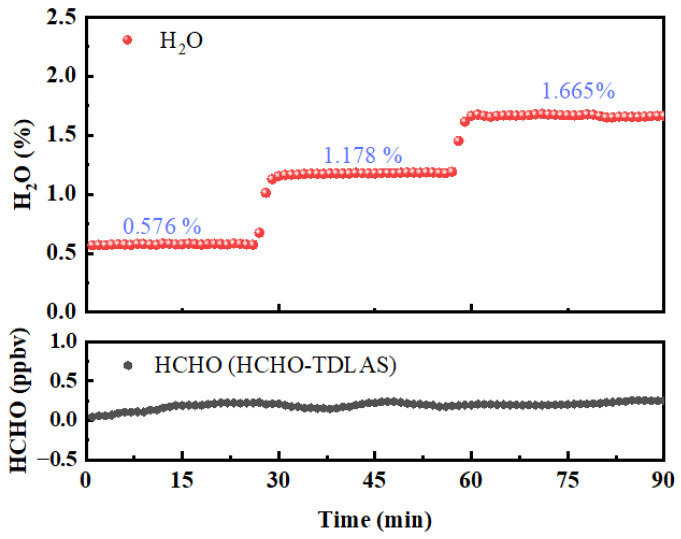
Test of HCHO–TDLAS against the interference of water vapor.

Although the HCHO absorption peak under the selected spectral line is not interfered by the water vapor absorption peak, there are still some water vapor absorption lines around 5.68 µm. In addition, compared with HCHO, the content of water vapor in the air is relatively higher, so it is necessary to further explore and analyze the influence of water vapor wings when detecting the concentration of HCHO under different levels of humidity. The configured high-purity N_2_, which has different contents of water vapor, was passed into the HCHO–TDLAS system to observe the changes in the concentration of the HCHO measured under different water vapor contents, and the results are shown in Figure 11. When the concentration of water vapor varies from 0.576% to 1.665%, the standard deviation of the concentration series of HCHO is only 45 pptv, indicating that the measurement results of the HCHO–TDLAS system are hardly affected by water vapor.

#### 3.9.3. Short-Term Comparison Experiments

**Figure 12:**
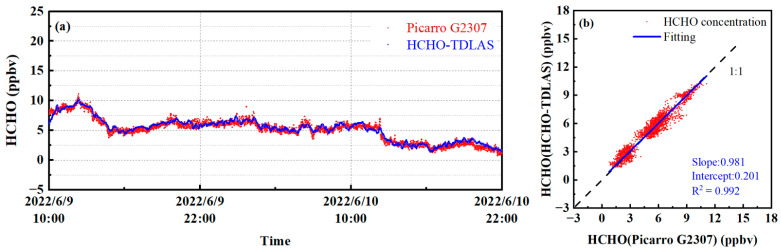
Comparison results: (**a**) results of 36 h continuous monitoring of the concentration of the HCHO gas in the atmosphere; (**b**) correlation analysis.

The HCHO–TDLAS, developed by ourselves, has been compared with the Picarro G2307 HCHO detection system (Picarro G2307 is a commercial instrument for measuring the concentration of HCHO gas based on triangular cavity CRDS technology, which has been applied to actual field observations by well-known institutions, such as the Environmental Protection Agency (EPA), Environment Canada, and the National Physical Laboratory (NPL) in the UK) through a short-term observation experiment, and the application ability of the HCHO–TDLAS in the actual atmospheric environment short-term monitoring was initially evaluated by comparing with Picarro G2307. When conducting the comparison experiment, the two devices used the same length of Teflon tube, and the vacuum pump from the outdoor air extraction. To prevent the dust in the atmosphere to cause pollution to the mirror, a 0.2 μm membrane filter was used to filter the air intake. The intake flow rate of HCHO–TDLAS was controlled at 600 mL/min through a mass flowmeter (CS200), and the cavity pressure was controlled at 30 Torr. The intake flow rate of the Picarro G2307 detector was 400 mL/min, and the cavity pressure was 100 Torr. To realize the parallel measurement, the intakes of the two devices were fixed together by cable ties. The time of the observation experiment was from 10:00 a.m. on 9 June 2022 to 22:00 a.m. on 10 June 2022. The 36 h measurement results of HCHO gas are shown in Figure 12, where the acquisition time of each HCHO concentration rate is 40 s.

The observation site is located in the suburbs. According to the results of measurement, the concentration of the HCHO showed an upward trend from 10:00 to 13:00 on June 9, and it began to decrease starting from 13:00. This is because the secondary source is usually the main source of HCHO in the atmosphere, which is the chemical oxidation process of the atmosphere. VOCs such as alkanes, alkenes, and the benzene series can be the precursors of the atmospheric photochemical process of HCHO under light conditions [41]. At noon on June 9th, the sunlight illumination was the strongest, and the concentration of HCHO peaked under the action of photochemistry. After 13:00, the light intensity decreased, so the concentration of HCHO also began to decline. The day of 10 June was cloudy and the atmospheric photochemical interaction was weak, so the concentration of HCHO remained at a low rate. The measurement results showed that the stability of HCHO–TDLAS was significantly better than that of Picarro G2307. This is consistent with the performance assessment of the Allan variance (127 pptv@40s for Picarro G2307 and 28 pptv@40s for HCHO–TDLAS). When the local concentration of HCHO changed obviously from 9:00 p.m. on 9 June to 10:00 a.m. on 9 June, HCHO–TDLAS could still reflect this changing trend in real-time. Correlation analysis was conducted on the two instruments, and the results are shown in Figure 12b. Under the response time of 40 s, the regression slope of (0.981 ± 0.002) was obtained, and the correlation R^2^ was 0.992. The regression slope deviation of the measurement results is about 2%, which may be caused by the measurement error of the HCHO–TDLAS and the Picarro G2307 (The uncertainty is 10%). To summarize, the two instruments have good consistency. The two HCHO detection instruments had good consistency. The results show that our system, HCHO–TDLAS, has satisfactory real-time monitoring capabilities for trace HCHO gas in the atmospheric environment.

### 3.10. Field Application

**Figure 13:**
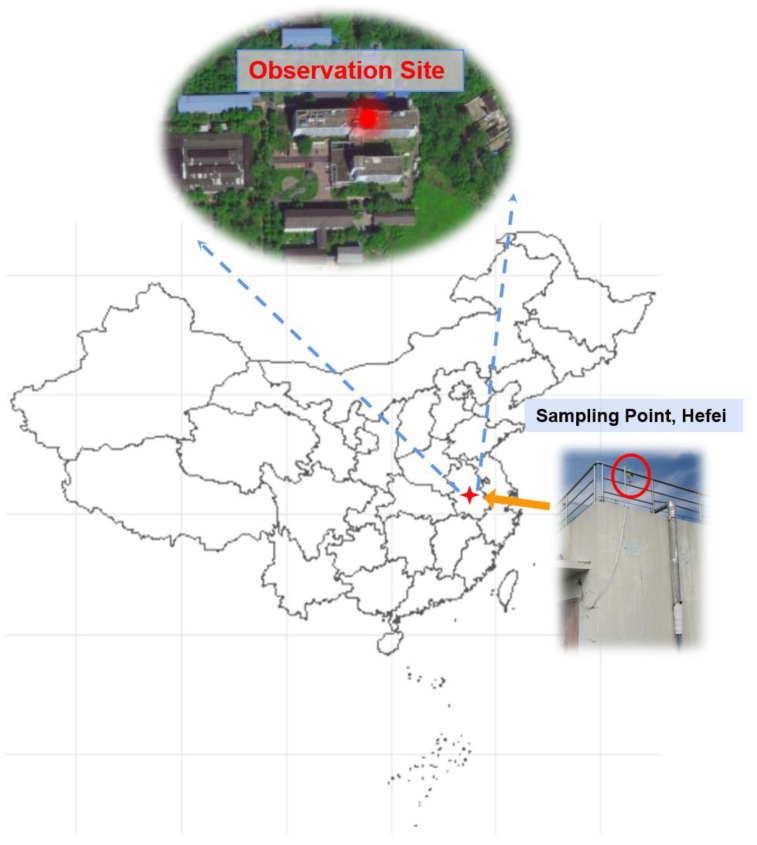
The observation site is located in the suburb of Hefei (longitude: 117°9′, latitude: 31°54′).

The instrument described here was deployed in an integrated field campaign in Hefei city from 9 September 2022 to 11 October 2022. The long-term field campaign was conducted at the Comprehensive Experimental Building of Anhui Institute of Optics and Fine Mechanics, Hefei Institute of Physical Science, Chinese Academy of Sciences (longitude: 117°9′, latitude: 31°54′) (Figure 13), and Picarro G2307 was also deployed in the activity. There is significant vegetation near the detection site. In addition, there is a road 200 m away from the detection site, and since the detection site is located on the outskirts of the city, the traffic flow on the road is usually low. In general, there are few pollution emission sources near the observation site, and the ambient air is relatively clean.

**Figure 14:**
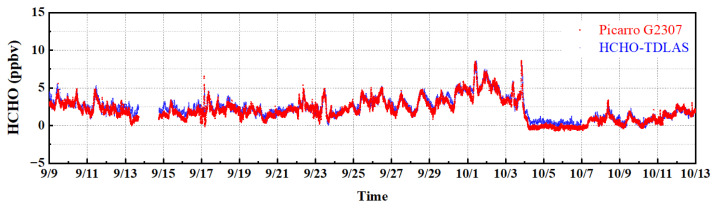
HCHO concentration sequence in an integrated field campaign.

The two detection systems are placed at a height of about 20 m above the ground. First, a Teflon sampling tube with anti-adsorption capability was installed at the air inlets of the two instruments, respectively. The two Teflon tubes were wrapped by a black heat insulation sleeve, which can not only prevent HCHO gas from being lost by photolysis during extraction and transmission but also prevent condensation on the inner wall of the intake tube. Second, 0.2 μm filters were installed at the Teflon sampling ports of both instruments, and the filters were changed every morning to prevent atmospheric dust and aerosol particles from affecting the measurements. In addition, since HCHO plays an important role in atmospheric photochemistry, and its concentration is related to the intensity of sunlight therefore, the air intake of the sample tube was fixed on the top floor with good lighting conditions. Figure 14 shows the comparison of field activity measurement results of the two instruments at 2 min acquisition time.

**Figure 15:**
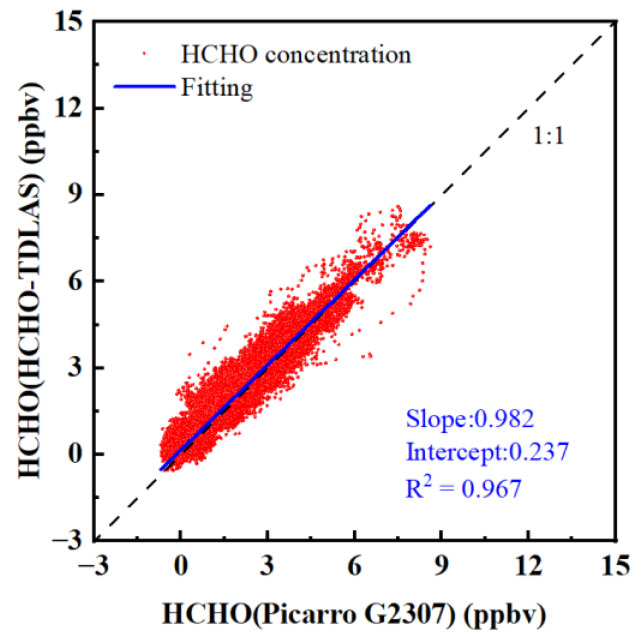
Correlation analysis.

According to the measurement results, the average HCHO concentration level during the observation period was low, and the overall trend of the measurement results of the two instruments was consistent. Rain occurred between 13 September and 14 September, and both instruments stopped measuring in order to prevent the instrument from flooding. There was a strong wind from 12 September to 16 September. The HCHO–TDLAS sampling tube, found on the day of 15 September, fell from the sampling position, resulting in the two sampling tubes being about 3 m apart, so there was some deviation in the measured values of the two instruments during this period. In order to compare the measurement results of the two instruments during rainfall, the sampling tubes of the two instruments were fixed, and rainproof devices were installed to ensure that the two instruments could continuously measure the concentration of HCHO in the atmosphere during rainfall. On 17 September, there were three peaks in HCHO concentration, which may be related to the sign painting construction in the north of the observation site that day. In addition, we observed that the HCHO concentration was at a high level under the action of atmospheric photochemistry during the period from 26 October to 3 October due to the strong daytime light on sunny days. The concentration of HCHO in the atmosphere dropped sharply after the rain in the early hours of 4 October. During the rain period from 4 October to 7 October, the HCHO concentration values measured by the two instruments were at a low level with relatively small fluctuations. The mean values of concentration of HCHO measured by Picarro G2307 and HCHO–TDLAS were −244 pptv and 363 pptv, respectively. The measurement results of Picarro G2307 were consistently lower than HCHO–TDLAS, this may be due to changes in the humidity of the air caused by rainfall (Picarro G2307 measurement and the inversion of HCHO concentration need to be empirically removed with linear post-fit corrections due to the residual cross-talk caused by water vapor) [42]. Correlation analysis was conducted on the concentrations of HCHO measured by two instruments, and the results are shown in Figure 15. According to the analysis results, the regression slope of concentration measured by the two instruments was 0.982, and the correlation R^2^ was 0.967. The results show that the HCHO–TDLAS instrument has a good ability to monitor ambient trace HCHO, in unattended continuous operation, for long periods of time.

## 4. Conclusions

In this work, a trace HCHO detection system (HCHO–TDLAS), based on the infrared tunable absorption spectrum of the quantum cascade laser, was developed and successfully applied to actual atmospheric environment monitoring. The suitable HCHO target absorption line and detection parameters were selected to effectively solve the interference problem in the field campaign. In order to suppress the interference noise between spots and improve the absorption path of gas, we designed a novel structure of a dual-incidence multi-pass cell. Compared with the direct absorption measurement, the system under the wavelength modulation scheme can achieve better detection performance. The background subtraction technique and wavelet digital signal processing algorithm were applied in the experiment of trace detection, and they can further improve the SNR of the system. The wavelength shift correction method can correct the signal position offset caused by wavelength shift. The method first calculates the deviation value of the signal position of the measured gas by the reference signal, and then, it adjusts the center position of 2f, in real-time, by software processing to suppress the signal and background measurement errors caused by wavelength drift. The detection sensitivity of 111 pptv (1σ) was achieved with 40 s acquisition time. The Kalman adaptive filtering technique was applied to the extraction of HCHO concentration, and the detection sensitivity was improved to 28 pptv (1σ), which meets the needs of trace HCHO detection in the atmospheric environment.

A comparative experiment of the Picarro G2307 HCHO detection system, based on CRDS technology, and HCHO–TDLAS was carried out for 36 h. The correlation of the measured concentration series was up to 0.992, and the slope was 0.981. The comparison results showed that the two systems had a good correlation. Moreover, the HCHO–TDLAS detection system was deployed to the long-term joint field observation in Hefei, Anhui Province from September to October 2022. The correlation with the observed concentration series of Picarro G2307 was as high as 0.967, with a slope of 0.982. The results show that the HCHO–TDLAS instrument has a good ability to monitor ambient trace HCHO in unattended continuous operation for long periods of time. In addition, the excellent performance and great application potential of the mid-infrared absorption line at 1759.73 cm^−1^, in the long-term observation of HCHO in the atmosphere, were also verified.

To further improve the detection performance of the HCHO–TDLAS, the instrument can be optimized from the following three aspects. Among these aspects, one is designing a more stable and suitable mechanical structure to further reduce the background drift caused by the insufficient stability of a mechanical structure, such as reducing the height of the mirrors and other optical components on the optical plate. The second aspect is designing a high-precision temperature control system to suppress the influence of temperature on the interference structure. In the third respect, the effective absorption optical path of the multi-pass cell is limited by the actual number of light spots distributed on the mirror’s surface. Therefore, to further improve the effective absorption optical path of the system and make it more suitable for high sensitivity detection, the dual-incidence structure can be improved into a multi-incidence structure. These three improvements are expected to further expand the application prospects of HCHO–TDLAS.

## Figures and Tables

**Table 1:** Comparison of the literature reported for the detection limit with some TDLAS systems.

Methods	Year	Laser Type	Time Resolution	Detection Limit (1σ)	Reference
TDLAS	2010	DFG	60 s	22 pptv	[22]
	2012	Lead salt	60 s	44 pptv	[24]
	2014	QCL	80–90 s	340 pptv	[29]
	2015	DFG	1 s	40 pptv	[18]
	2015	ICL	90 s	3 ppbv (precision of 69 pptv)	[31]
	2016	ICL	40 s	73 ppbv	[33]
	2018	ICL	1 s	3.2 ppbv	[35]
	2019	ICL	10 s	51 pptv	[32]
	2022	QCL	40 s	28 pptv	This work

## Data Availability

The data presented in this study are available on request from the corresponding author.
